# Laser-stimulated human gingival fibroblasts: alterations in migration, secretome production, and induction of reactive oxygen species

**DOI:** 10.1007/s10103-025-04499-4

**Published:** 2025-06-09

**Authors:** Lina María Escobar, Marggie Grajales, Zita Bendahan, Catalina Arango, Paula Baldión

**Affiliations:** 1 Grupo de Investigaciones Básicas y Aplicadas en Odontología, IBAPO Facultad de Odontología, Universidad Nacional de Colombia, Bogotá, Colombia., Bogotá, Colombia; 2 Unidad de Manejo Integral de Malformaciones Craneofaciales UMIMC, Facultad de Odontología, Universidad El Bosque, Bogotá, Colombia., Bogotaá, Colombia; 3 Departamento de Salud Oral, Facultad de Odontología, Universidad Nacional de Colombia, Bogotá, Colombia, Bogotá, Colombia

**Keywords:** Gingival fibroblasts, Laser, Migration, Mitochondrial membrane potential proliferation, Reactive oxygen species, Secretome

## Abstract

This study aimed to investigate the effects of low-level laser irradiation on human gingival fibroblasts, specifically examining changes in proliferation, morphology, migration, and reactive oxygen species (ROS) production. Additionally, we assessed the impact of the secretome from irradiated fibroblasts on non-irradiated cell proliferation and migration. Human gingival fibroblasts were exposed to 650–940 nm laser irradiation for 50 s. Cell proliferation was quantified using resazurin, while migration was evaluated through a wound generation assay and by treating non-irradiated cells with the secretome from irradiated fibroblasts. We also analysed changes in type I collagen (COL1A1) expression, ROS production, and mitochondrial membrane potential (ΔΨm). Both 650 nm and 940 nm laser treatments induced morphological changes and significantly enhanced cell proliferation, observed 10 days post-irradiation, without causing cell detachment or death. Non-irradiated cells treated with the secretome from 940 nm-irradiated fibroblasts exhibited increased cell density at five and 10 days post-irradiation. Laser treatment at both wavelengths significantly stimulated cell migration. Cells irradiated with the 650 nm laser showed increased COL1A1 expression at five days, while those treated with 940 nm demonstrated a marked increase at 10 days. Low-level laser treatment led to significant increases in both ROS production and ΔΨm. In conclusion, low-level laser treatment induced morphological changes and increased proliferation, cell migration, and COL1A1 expression in gingival fibroblasts. Treatment with laser-stimulated cell secretome significantly enhanced gingival fibroblast proliferation. The 940 nm laser treatment elicited the most pronounced cellular changes, particularly in relation to increased ROS production and ΔΨm.

**Graphical abstract:**

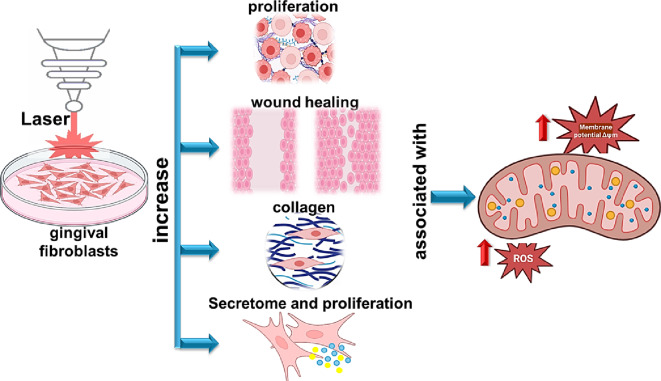

## Introduction

Photobiomodulation (PBM) using red and near-infrared wavelengths (600–1000 nm) has consistently demonstrated favourable dose-dependent effects on cell proliferation [1, 2]. Low-level laser therapy (LLLT) achieves these effects by delivering an optimal stimulatory dose, which triggers mitochondrial polarisation and enhances membrane permeability [3]. The energy emitted by photons is absorbed by cell membrane photoreceptors and mitochondrial electron transport chain molecules, initiating a cascade of chemical reactions, signalling pathways, and cellular responses [4]. These include ATP synthesis, alterations in cell membrane permeability, the generation of reactive oxygen species (ROS), and nitric oxide release [5].

LLLT also influences intracellular pH by modifying the concentration gradient of H+, activating ATPases that regulate proton flow back into the mitochondrial matrix, thus altering the electrochemical proton gradient [6]. These shifts lead to increased intracellular calcium levels, stimulating cellular metabolism and promoting RNA and DNA synthesis, as well as the secretion of proteins, growth factors, and cytokines [7, 8]. Collectively, these mechanisms enhance extracellular matrix synthesis, modulate the inflammatory response [9], and support local immune, vascular, and nervous system functions [10], allowing cells to perform more efficiently [11]. These processes enhance cell viability, growth, proliferation, and tissue healing [12].

The secretome, a bioactive medium secreted by cells, is an emerging focus in regenerative medicine [13]. Comprising primarily proteins, lipids, and extracellular vesicles, the secretome contains paracrine factors with trophic, cytoprotective, and immunomodulatory properties. It has been shown to accelerate wound healing, re-epithelialisation, and collagen production while reducing inflammation [13–15]. Ahangar et al. (2020) [13], identified cytokines, growth factors [(Fibroblast Growth Factor 2 (FGF-2), Vascular Endothelial Growth Factor (VEGF)], and accessory molecules in the gingival fibroblast secretome that contribute to effective wound healing in experimental models. Bakopoulou et al. (2017) [14], highlighted the role of gingival tissue-derived mesenchymal stem cell (MSC) secretomes in keratinisation, ectodermal development, and chemotaxis, which are crucial for regenerative processes. However, limited research has explored how LLLT impacts the secretome and its potential to enhance tissue regeneration.

Periodontal diseases and gingival injuries present significant clinical challenges that often necessitate innovative approaches for tissue regeneration [16]. While PBM has shown potential in enhancing gingival fibroblast proliferation, migration, and extracellular matrix synthesis, the variability in laser parameters and protocols complicates its standard*isa*tion for clinical use [17]. Moreover, the role of secretomes derived from laser-treated cells in influencing the behaviour of untreated cells remains underexplored. Investigating these areas is essential to better understand and optimise LLLT for clinical applications [18].

The dual potential of LLLT in directly enhancing cellular functions and modulating the secretome offers an integrated approach to improving tissue repair [19]. Gingival fibroblasts, which play a central role in maintaining periodontal health and wound healing, are ideal targets for such interventions [20]. By elucidating the mechanisms through which LLLT impacts both fibroblast activity and secretome composition, this study attempts to advance the clinical application of laser-based therapies, fostering more efficient and precise tissue regeneration.

This study aims to achieve two primary objectives: (1) to evaluate the effect of laser irradiation on the proliferation, gene expression, and migration of gingival fibroblasts, and (2) to investigate the impact of the secretome secreted by laser-irradiated gingival fibroblasts on the proliferation and migration of non-irradiated cells.

## Methods

### Isolation and primary culture of human gingival fibroblasts

This quantitative in vitro experimental study analysed human gingival fibroblasts obtained from gingival explants of patients over 18 years old undergoing periodontal surgeries. The study was approved by the institutional ethics committee (CIEFO-094-2023), and participants provided informed consent before tissue collection.

Gingival fibroblasts were isolated using a standard*ise*d procedure. Briefly, small tissue fragments were dissociated with a mixture of collagenase (100 U/mL) and dispase (1 mg/mL) for one hour, washed with fresh medium, and centrifuged. The resulting cell pellet was seeded into 25 cm² culture flasks and cultured for seven days at 37 °C in a 5% CO_2_ incubator. Cells were maintained in Roswell Park Memorial Institute (RPMI) 1640 medium (Hyclone, Thermo Scientific) supplemented with 10% foetal bovine serum (FBS) (Gibco, Thermo Fisher Scientific) and antibiotics (100 U/mL penicillin + 100 µg/mL streptomycin) until reaching 70% confluence.

Cells were then dissociated using 0.25% trypsin, counted, seeded, and treated with low-level laser. Gingival fibroblasts were character*ise*d by their spindle-shaped morphology. Immunostaining for vimentin and actin (phalloidin) was performed to confirm fibroblast identity. Flow cytometry revealed high expression of CD63 and CD10 and low expression of CD24 and CD326, further confirming the gingival fibroblast population [21].

### Treatment of gingival fibroblasts with low-level laser

Laser application was performed on gingival fibroblast cultures using two diode lasers: a 650 nm (visible red) LX 16 Woodpecker diode laser (Guilin Woodpecker Medical Instrument CO.) and a 940 nm (near-infrared) Biolase Epic X (Biolase). Devices were calibrated using an OPHIR NOVA II optical power meter P/N 7Z01550 (Ophir Optronics). Laser characteristics and application parameters are shown in Tables 1 and 2, following the protocol suggested by Jenkins and Carroll [22] (Table 3).

**Table 1:** Technical specifications of laser equipment

Wavelength	650 ± 20 nm	940 ± 10 nm^*^
Manufacturer	Guilin Woodpecker Medical Instrument Co., Ltd.	Biolase
Model Identifier	LX 16 Plus	Epic X
Place of Manufacture	Guangxi– China	California– USA
Emitter type	Diode GaAlAs laser	Diode InGaAs laser
Laser beam delivery system	Fibre optic	Fibre optic
Operating mode	Continuous-wave operation (CW operation) Settings: L1 mode	Continuous-wave operation (CW operation) Settings: Pain Therapy mode. Deep tissue handpiece– no spacer
Laser beam profile	Gaussian	Gaussian
Maximum output power	0,2 W ± 20%	10 W ± 20%

* Guidelight 625–670 nm

**Table 2:** Application parameters of the study

Wavelength	650 nm	940 nm
Beam spot size (cm^2^)	0.38	0.78
Area irradiated per application (cm^2^)	1	1
Area per well (96/ 12)* (cm^2^)	0.32/ 4	0.32/ 4
Power density (W/cm^2^)	0.26	0.13
Power output (W)	0.1	0.1
Exposure time (s)	50	50
Energy density (J/cm^2^)	13.15	6.4
Energy per point (J)	5	5
Total energy per well (96)* (J)	5	5
Total energy per well (12)* (J)	20	20
Application points per well (96)*	1	1
Application points per well (12)*	4	4
Application technique	Fixed-point with support	Fixed-point with support
Distance from laser spot (96/ 12*) (cm)	1.1/ 1.9	1.1/ 1.7
Number of applications	Single-dose	Single-dose

* Calculations performed for 96-well and 12-well cell culture plates, respectively

Cells were seeded in multiwell plates and divided into three groups: a control group (no laser irradiation) and two experimental groups treated with either 650–940 nm laser for 50 s. The power output was 0.1 W for both wavelengths. Doses were calculated per square centimetre (cm²). Application parameters were based on a previous systematic review [23] and prior research group results [24]. A single laser application was performed for each condition. The irradiation distance was calculated from the laser beam exit to the well bottom, avoiding contact between the laser tip and well sides to prevent contamination.

For cell viability, proliferation, and migration analysis, 12-well culture plates with 5,000 cells/well were used. For proliferation and migration studies of cells treated with laser-stimulated secretome, 6-well plates with 100,000 cells/well were used. Reactive Oxygen Species (ROS) quantification and mitochondrial membrane potential (Δψm) evaluation used 96-well plates with 10,000 cells/well.

**Fig. 1:**
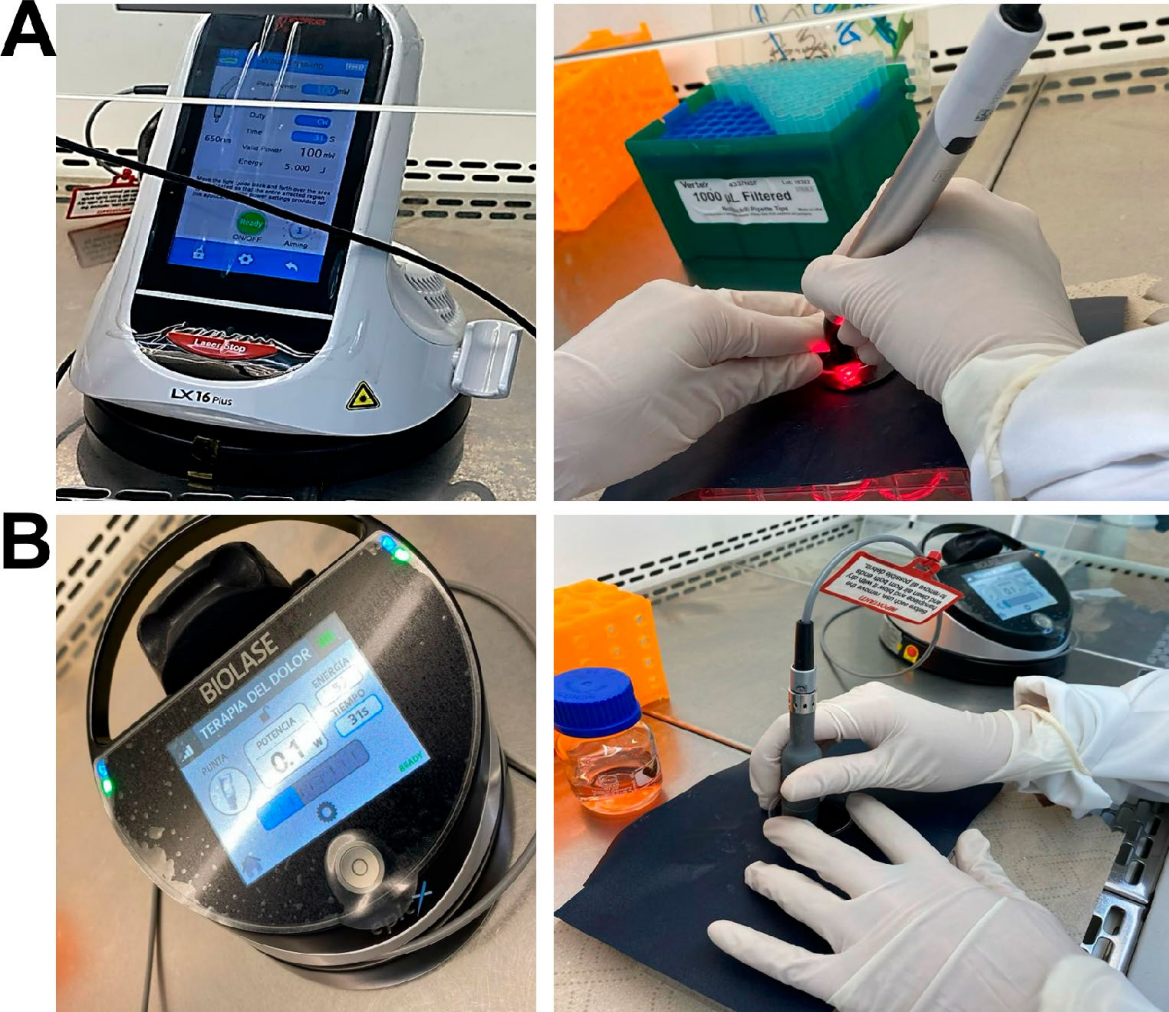
Application of laser on human gingival fibroblasts. Cells seeded in multiwell plates without culture medium were subjected to a single laser application, using a black plastic sheet to avoid scattering of the emitted laser light to adjacent wells, as shown in the photographs. A wavelength of 650 nm was applied with (**A**) the LX 16 Woodpecker diode laser device and a wavelength of 940 nm with (**B**) the Biolase Epic X diode laser

During laser application, culture plates were uncovered and the medium was removed. Fresh medium was promptly applied after irradiation. Conversely, the plates were covered with a black plastic sheet to prevent the scattering of emitted laser light to adjacent wells. Only the designated well was left uncovered for laser irradiation, with a hole in the plastic cover matching its diameter, ensuring the application of parameters per square centimeter (Fig. 1).

### Evaluation of viability, proliferation, and morphological changes of gingival fibroblasts exposed to two laser wavelengths

Fibroblast viability and proliferation, with or without LLLT, were assessed using trypan blue dye exclusion in a haemocytometer and fluorometric resazurin assay. Trypan blue selectively labels dead cells, as it cannot penetrate the intact membranes of live cells. Under light microscopy, only dead cells appear blue. Resazurin, a non-fluorescent compound, is irreversibly reduced to highly fluorescent resorufin, with the increase proportional to mitochondrial activity in viable cells. Cells seeded in multiwell plates were treated with resazurin solution (4.4 µg/well) and incubated at 37 °C for four hours. Fluorescence intensity was then measured using a Tecan Infinite M2000 Pro reader (Tecan, Männedorf, Switzerland) at 650 nm and 940 nm wavelengths [25].

Daily morphological changes were observed via phase contrast light microscopy and compared to untreated cells to determine LLLT-induced phenotypic alterations. Photographic documentation continued for 10 days post-irradiation using a Zeiss Axio Imager M2 inverted microscope and AxioCam Hrm camera (Zeiss, Germany).

### Migration analysis of gingival fibroblasts treated with low-level laser

For migration analysis, 5,000 gingival fibroblasts were seeded per well in 12-well plates. After 24 h of adhesion, a cell-free area was created in each well using a 200 µl pipette tip [26]. The culture medium was removed, and cells were irradiated with 650 nm and 940 nm lasers for 50 s. Three photographs of the wound area were taken per well at 24 h, 5-, 7-, and 10-days post-irradiation. Wound closure was quantified using ImageJ software and expressed as a percentage of area closure compared to non-irradiated control cells.

### Proliferation and migration of gingival fibroblasts treated with secretome from laser-stimulated cells

Secretome or conditioned medium was obtained from human gingival fibroblasts 72 h after a single 50 s irradiation with 650–940 nm laser. This time duration was selected due to evidence of morphological changes in the cells. For secretome collection, 100,000 cells/well were seeded in six-well plates. After 24 h of adhesion, laser treatment was performed. The culture medium was collected, cell debris removed by centrifugation at 800 g for 5 min, filtered through a 50 mm membrane, and stored at 4 °C for use on non-irradiated gingival fibroblasts. This medium was replaced every 48 h [13].

Gingival fibroblast migration was analysed over 10 days, with images taken at 24 h, 5-, 7-, and 10-days post-irradiation for directly irradiated cells, and at 5 and 10 days for secretome-stimulated cells. For this analysis, 100,000 cells/well were seeded in six-well plates. Each well was divided into two zones, one of which was blocked with a polyvinyl chloride surgical tape (Micropore, 3 M, St Paul, Minn, USA) to prevent cell adhesion in that area. After 24 h of adhesion, the surgical tape was removed, and the cells were exposed to secretome from 650 to 940 nm laser-treated cells, or the control group (cells without laser exposure) [27].

Cell counting was performed using the method described by Germundsson & Lagali [28] with ImageJ software (National Institutes of Health, MD, USA). Images of the previously tape-covered area were cropped to 400 × 400 mm, thresholded using auto-default settings, converted to binary, and processed with the “watershed” function to separate cells. The “analyze particles” function was used for automated cell counting. Results are expressed as cells per mm² from three independent photographs of each experimental group.

### Evaluation of changes in collagen I gene expression

Total RNA was extracted using the Quick-RNA MicroPrep Kit (Zymo Research) from 10,000 cells/well seeded in 12-well plates and subjected to 650–940 nm laser treatment for 50 s, 24 h after adhesion. Complementary DNA (cDNA) was synthes*ise*d using the ProtoScript II First Strand cDNA Synthesis kit (New England BioLabs, MA, USA), with samples incubated at 25 °C for 5 min and then at 42 °C for 60 min. RT-qPCR was performed to evaluate Collagen type I (COL1A1) gene expression using the Luna Universal qPCR Master Mix kit (New England BioLabs) with SYBR Green I and quantified on a BioRad CFX96 Real-Time System thermal cycler. Glyceraldehyde-3-phosphate dehydrogenase (GAPDH) served as the reference gene (Table 3).

**Table 3:** Characteristics of the primers used in RTqPCR

Gen	Forward primer	Reverse primer	Amplicon size (bp)
*COL 1A1*	5’-TGACCTCAAGATGTGCCACT − 3’	5’-ACCAGACATGCCTCTTGTCC − 3’	197
*GAPDH*	5’-GAAGGTGAAGGTCGGAGTC-3’	5’GAAGATGGTGATGGATTTC-3’	226

Relative gene expression changes were analysed using Schefe’s method [29], comparing each treatment group to the untreated control and normalizing to GAPDH expression. Control sample expression was set to one, with changes greater than twofold considered significant. PCR amplification efficiencies were calculated using LinRegPCR (Academic Medical Center, AMC, Amsterdam, Netherlands).

### Evaluation of ROS production in gingival fibroblasts treated with LLLT

Reactive oxygen species (ROS) production induced by 650 nm and 940 nm laser exposure for 50 s was quantified using the intracellular oxidation probe 2’,7’-dichlorodihydrofluorescein diacetate (H2DCF-DA, 25 µM; Sigma, Merck, St. Louis, MO, USA). In the presence of ROS, H2DCF-DA oxid*ise*s to the highly fluorescent compound 2’,7’-dichlorofluorescein (DCF). Gingival fibroblasts were incubated with H2DCF-DA in the dark for one hour at 37 °C in a 5% CO_2_ atmosphere. Cells were then washed and exposed to tert-butyl hydroperoxide (TBHP, 1X) as a positive control for ROS generation, ascorbic acid (AA, 50 µg/mL) as a negative control, or TBHP + AA as an ROS production inhibitor for 30 min following LLLT irradiation [30, 31]. Fluorescence intensity was measured using a Tecan Infinite M2000 Pro spectrophotometer (excitation 490 nm, emission 520 nm) to quantify ROS levels in treated versus untreated cells.

In parallel, cells seeded on glass slides in 24-well plates were subjected to the same conditions and imaged using a Zeiss Axioimager A2 fluorescence microscope (Göttingen, Germany) to visual*ise* ROS production.

### Evaluation of mitochondrial membrane potential changes in LLLT-treated gingival fibroblasts

Mitochondrial membrane potential (Δψm) was assessed using the Tetramethylrhodamine Ethyl Ester (TMRE) probe (Abcam, Cambridge, UK) according to the manufacturer’s instructions. Gingival fibroblasts were seeded in dark 96-well plates and allowed to adhere for 24 h. Following exposure to 650–940 nm LLLT for 5 s, TMRE solution was added to a final concentration of 200 nM and incubated for 30 min at 37 °C in the dark. TMRE, a positively charged molecule, accumulates in active mitochondria due to their high negative charge. In depolari*se*d or inactive mitochondria, TMRE is not retained, resulting in decreased fluorescence.

Carbonyl cyanide-p-trifluoromethoxyphenylhydrazone (FCCP, 20 µM) was used as a positive control for mitochondrial depolarisation and was added 10 min before TMRE staining. Fluorescence intensity was measured using an Infinite M200 spectrofluorometer (Tecan, Männedorf, Switzerland) at excitation 549 nm and emission 575 nm [24].

### Data analysis

Descriptive statistics, including measures of central tendency and dispersion, were calculated for all response variables across experimental groups. Comparisons between groups were performed using one-way ANOVA followed by Tukey’s HSD post hoc test, assuming homogeneous variances (Levene’s test). When appropriate, the Kruskal-Wallis test with Dunn’s multiple comparisons was used. Normal distribution was verified using the Shapiro-Wilk test. All analyses were conducted using IBM SPSS 27 software (SPSS, Chicago, IL, USA) and a significance level of *P* < 0.05 was considered statistically significant between experimental groups.

## Results

### LLLT stimulates proliferation and induces time-dependent morphological changes in gingival fibroblasts

**Fig. 2:**
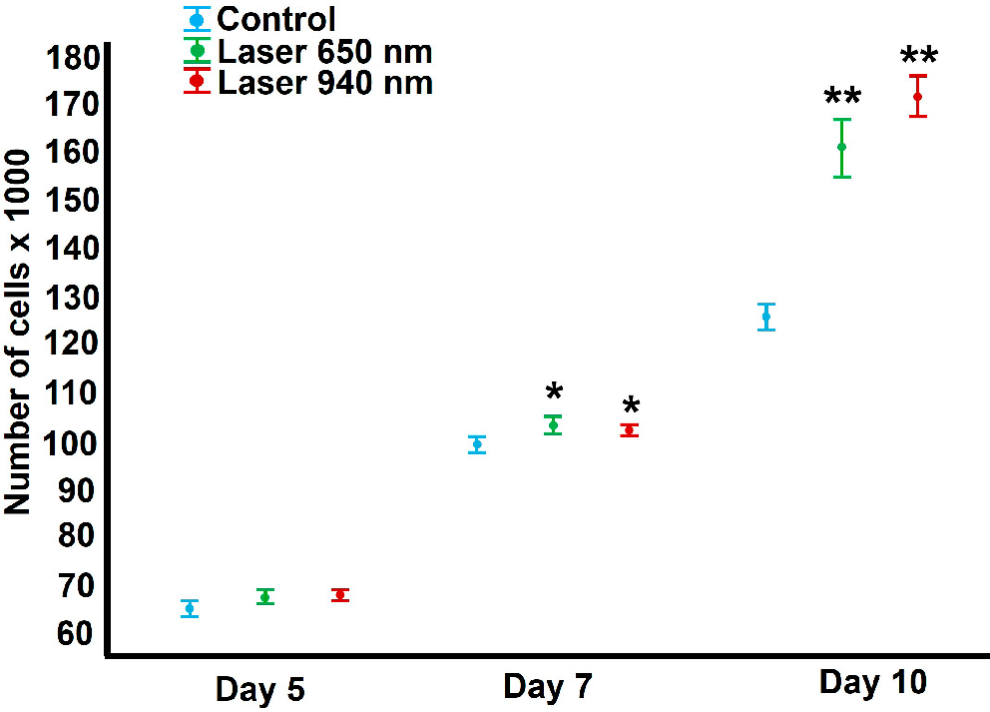
Effect on cell proliferation induced by LLLT. Treatment with 650 nm and 940 nm laser for 50 s produced a significant increase in cell number when compared to the group of cells without laser irradiation (Control) only at seven- and 10-days post-irradiation. No significant changes were determined at the other times. Data were analysed using a one-way ANOVA followed by Tukey’s HSD post hoc test, assuming homogeneous variances (Levene’s test). Statistically significant differences between treated and untreated control groups are indicated by asterisks *(*p* < 0.05), ** (*p* < 0.01). Results are presented as the mean and confidence interval (CI) from two independent experiments with three replicates each (*n* = 6)

Cell number and proliferation analysis revealed no significant differences between the 650 nm and 940 nm laser-treated groups compared to the non-laser-treated control group at five days post-irradiation. However, by day seven and 10 post-irradiation, a significant increase in cell number was observed in fibroblasts irradiated with both 650 nm and 940 nm wavelengths compared to the control group. No significant differences were found between the two wavelengths at any time point evaluated (Fig. 2). Cell viability remained consistent across all experimental groups throughout the study period (data not shown).

**Fig. 3:**
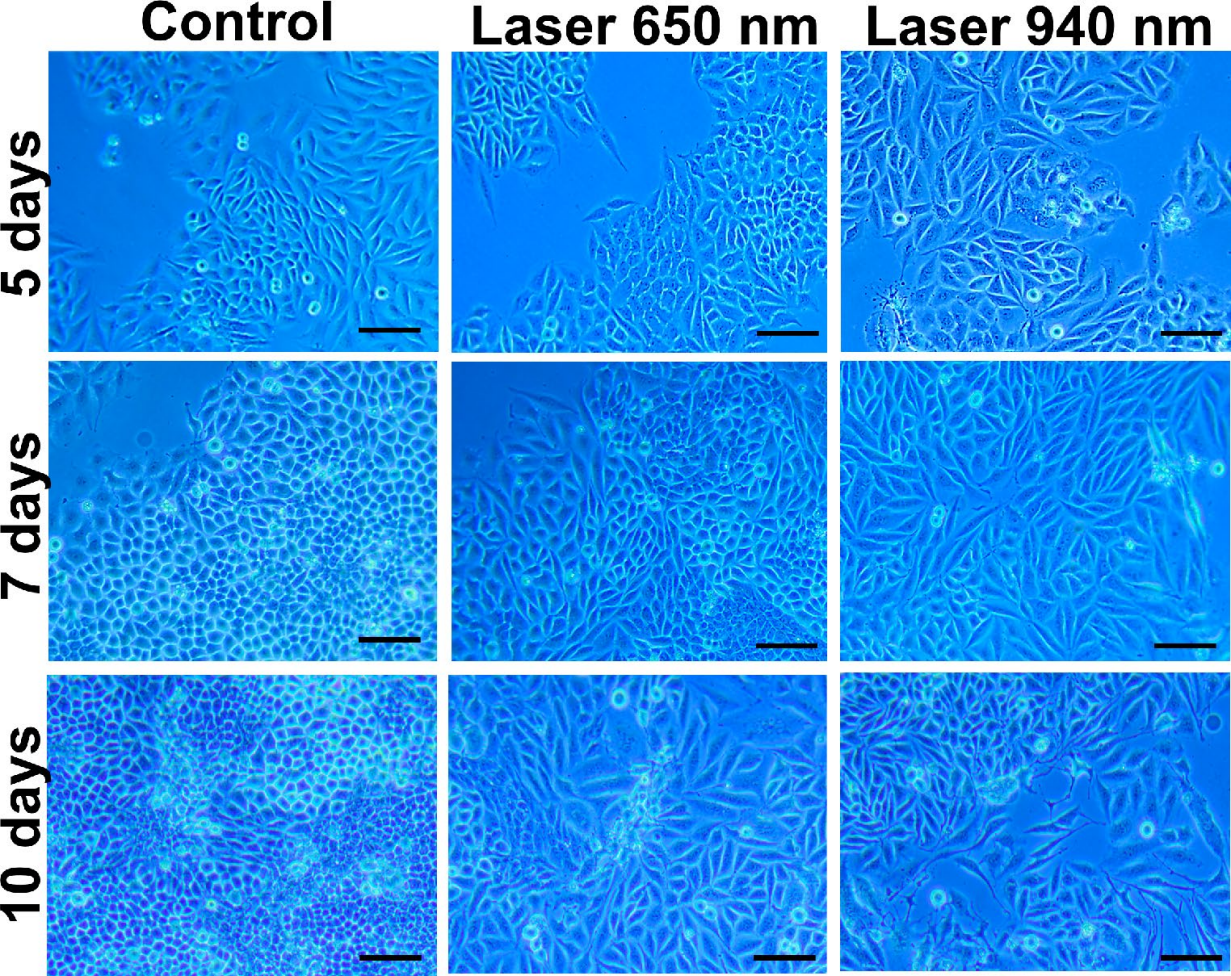
Human gingival fibroblasts treated with lower-level laser. Cells were treated with 650 nm and 940 nm laser for 50 s and were maintained in culture for 10 days post-irradiation. The control group corresponded to cells without irradiation. Larger cells and cell prolongations were observed when the laser was applied compared to the control group. Magnification bar: 200 μm

Morphological changes in gingival fibroblasts were analysed 10 days after treatment with 650 nm and 940 nm lasers for 50 s. As shown in Fig. 3, LLLT induced morphological alterations, characterised by larger cells and more extensive cytoplasmic prolongations, observable from five days post-irradiation for both wavelengths. These changes were more pronounced at 10 days post-irradiation, with the most notable morphological differences observed in cells treated with the longer wavelength laser (940 nm). No cell detachment or death was evident in any experimental group (Fig. 3).

### Treatment with laser-stimulated cell secretome significantly increases gingival fibroblast proliferation

**Fig. 4:**
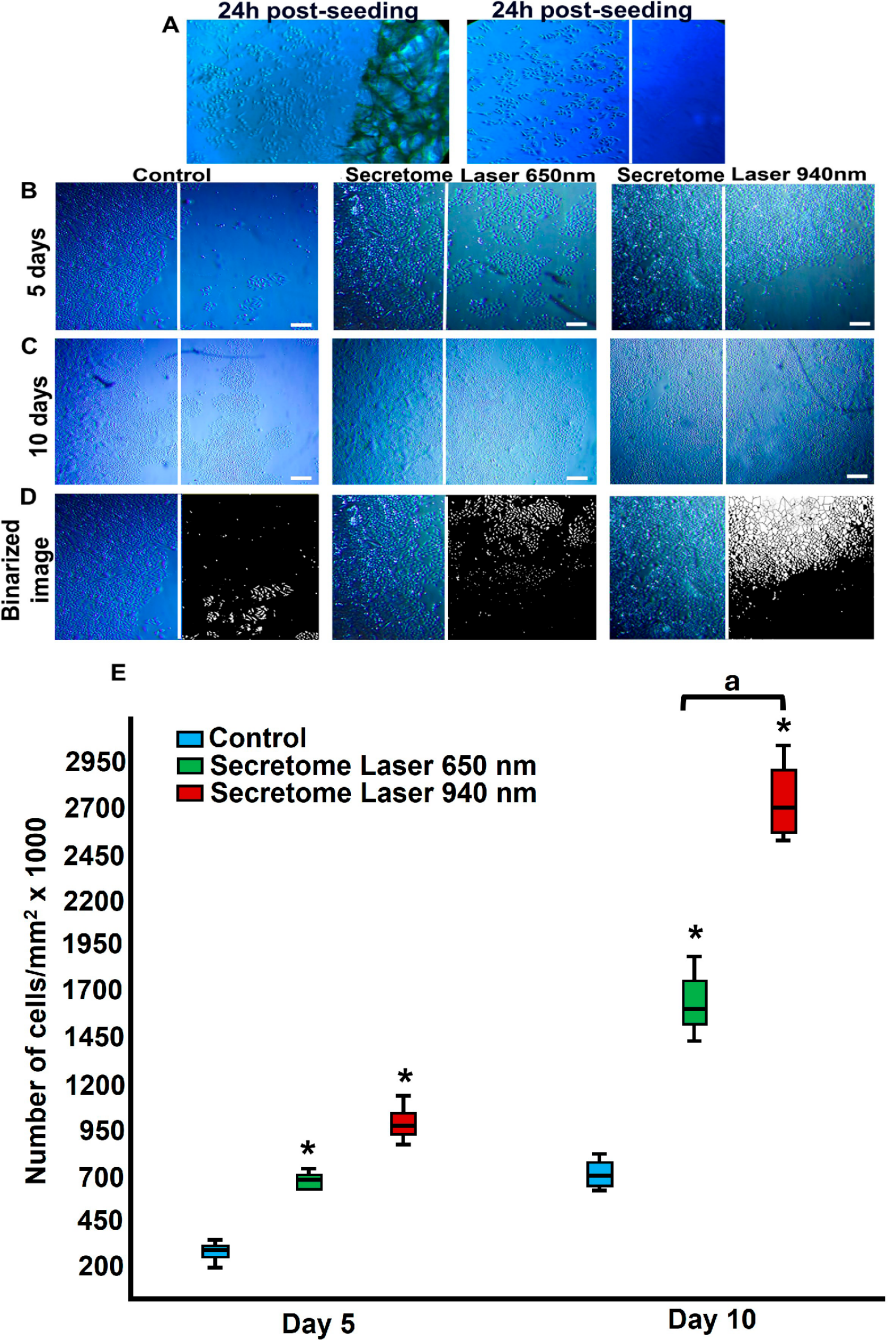
Changes in the number of gingival fibroblasts induced by secretome treatment. Cells were seeded in a 6-well plate by dividing the well into two parts by adhering surgical tape (Micropore), which was removed at 24 h post-seeding (**A**). These cells were exposed to secretome obtained from 650 nm and 940 nm laser-treated cells. The results of secretome treatment were evaluated at five (**B**) and 10 days (**C**) by the ImageJ program using binary images that allowed cell counting (**D**). The white line corresponds to the boundary zone of the previously adhered surgical tape (Micropore). Changes in cell number were evaluated at five days and 10 days of culture and compared with the group of cells that were not treated with secretome (Control) (**E**). The data were compared within the same days, using the Kruskal-Wallis H test followed by Dunn’s multiple comparisons test. Statistically significant differences between treated and untreated control groups are indicated by asterisks *(*p* < 0.05), ** (*p* < 0.01). Lowercase letters (a) show significant differences between test groups. Results are presented as the median and interquartile range from two independent experiments with three replicates each (*n* = 6). Magnification bar: 100 μm

The effects of the secretome from laser-treated gingival fibroblasts on cell proliferation and migration were assessed using a 6-well plate assay. Wells were divided into two sections using surgical tape (Micropore), which was removed 24 h post-seeding (Fig. 4A). As shown in Fig. 4 (B, C), notable increases in cell density were observed at five and 10 days in groups treated with secretome derived from cells exposed to 650 nm and 940 nm laser treatments. The most substantial increase in cell proliferation was evident in cells treated with secretome from fibroblasts exposed to the 940 nm laser, compared to the untreated control group. Cell counting was performed using binary images analysed with ImageJ software (Fig. 4D). The increase in cell number following stimulation with secretome from laser-treated fibroblasts was statistically significant at both five and 10 days, with the most pronounced changes observed in the group exposed to secretome from 940 nm laser-treated cells (*P* < 0.01) (Fig. 4E).

### LLLT treatment enhances cell migration and wound closure

**Fig. 5:**
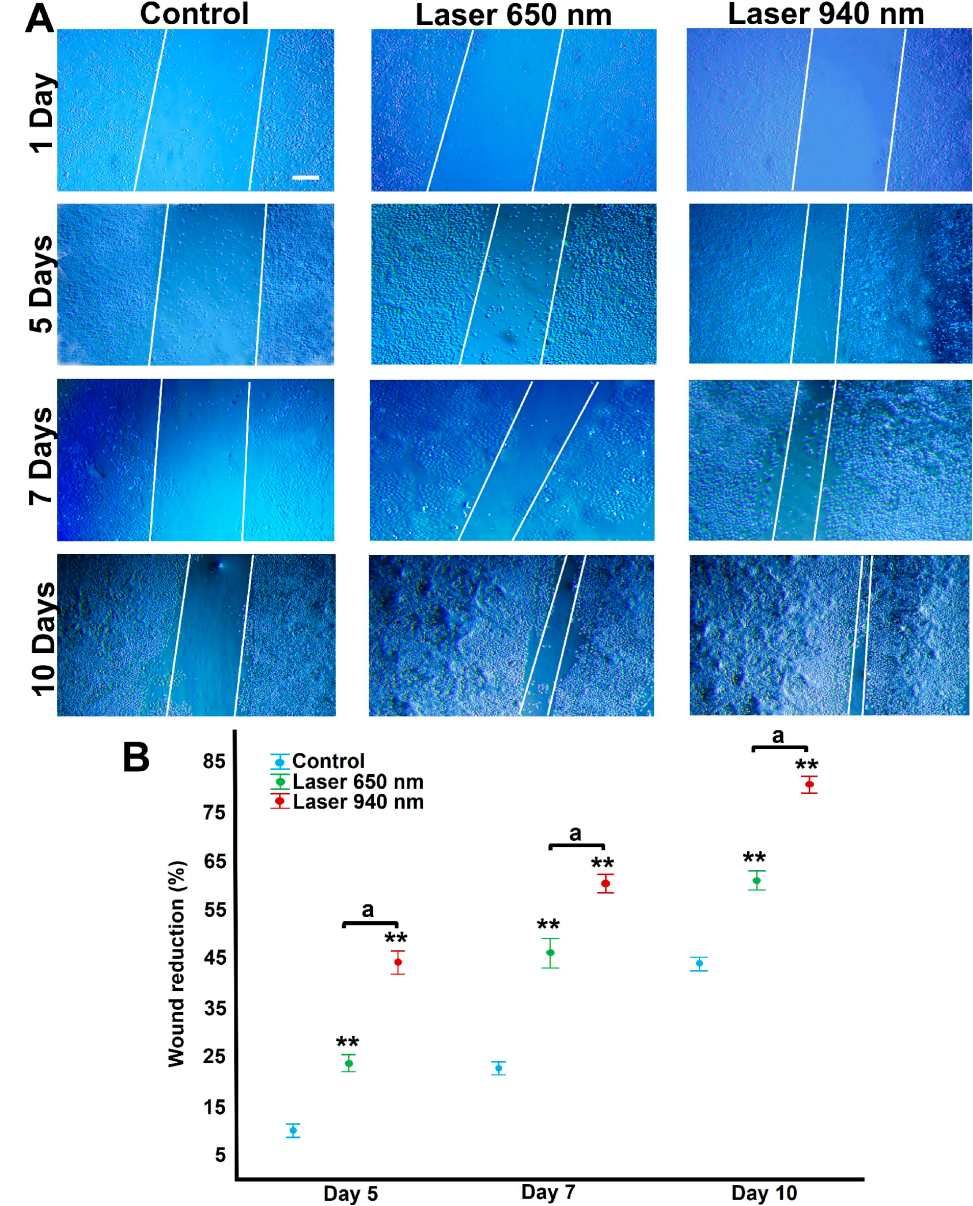
Cell migration and proliferation assay. (**A**) Representative photomicrographs of wound closure by fibroblasts after treatment with 650 nm and 940 nm laser at 24 h, five-, seven-, and 10-days post-irradiation are shown. White lines correspond to previously generated wound boundaries. Magnification bar: 100 μm. (**B**) The percentage of wound reduction was higher in cells treated with 940 nm laser at all times analysed (five-, seven-, and 10-days post-irradiation). The control group corresponds to non-irradiated cells. The data were compared within the same days, using a one-way ANOVA followed by Tukey’s HSD post hoc test, assuming homogeneous variances (Levene’s test). Statistically significant differences between treated and untreated control groups are indicated by asterisks *(*p* < 0.05), ** (*p* < 0.01). Lowercase letters (a) show significant differences between test groups. Results are presented as the mean and confidence interval (CI) from two independent experiments with three replicates each (*n* = 6)

Both 650 nm and 940 nm wavelengths stimulated cell migration and wound closure, with effects becoming apparent from day five post-irradiation. The effect was most pronounced 10 days after treatment with the 940 nm laser for 50 s (Fig. 5). At this time point, wound closure reached 75% in fibroblasts treated with the 650 nm wavelength and 84% (*P* < 0.01) with the 940 nm wavelength, compared to 54% (*P* < 0.05) in the non-laser-treated control group. The differences in closure percentage between all three experimental conditions were significant, with fibroblasts treated with the 940 nm laser for 50 s exhibiting the greatest cell migration (Fig. 5).

### Laser-treated gingival fibroblasts show increased COL1A1 expression

**Fig. 6:**
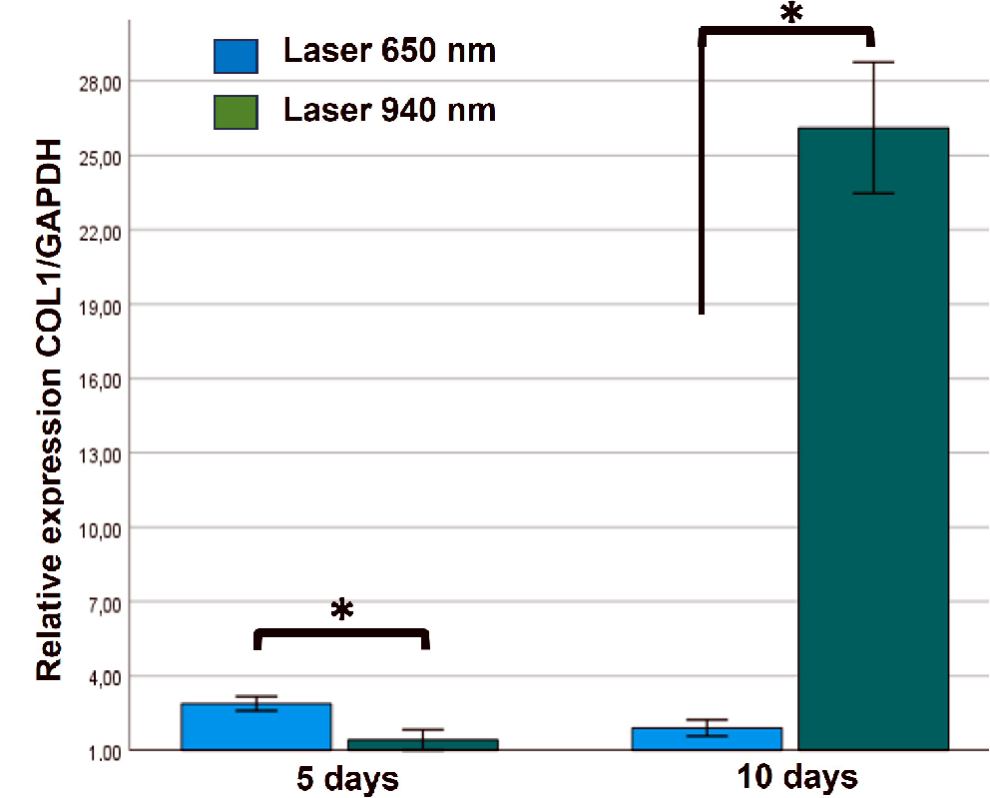
Relative expression of COL1A1 in human gingival fibroblasts treated with Laser of 650 nm and 940 nm at five and 10 days. COL1A1 transcripts were quantified by RT-qPCR considering the transcript expression value of unexposed cells (baseline expression = 1). Data are expressed relative to glyceraldehyde 3-phosphate dehydrogenase (GAPDH) gene expression levels. The data were analysed within the same days using the Student’s t-test for independent samples, after verifying compliance with the assumption of normal distribution with the Shapiro Wilk statistic and homoscedasticity of variances with the Levene test. The P value less than 0.05 was statistically significant (*). Data are shown as the mean ± SD of two independent experiments in duplicate (*n* = 6)

Human gingival fibroblasts treated with 650–940 nm lasers were evaluated for changes in COL1A1 gene expression as a marker of metabolic activity, extracellular matrix production, and fibroblast-mediated healing. Relative gene expression analysis revealed a 2.7-fold increase in COL1A1 expression in fibroblasts treated with the 650 nm laser for five days (*P* < 0.05). At 10 days post-irradiation with the 940 nm laser, a significant 23.2-fold increase in relative COL1A1 expression was observed (*P* < 0.01) (Fig. 6).

### Cellular effects induced by laser are associated with increased ROS and Δψm

**Fig. 7:**
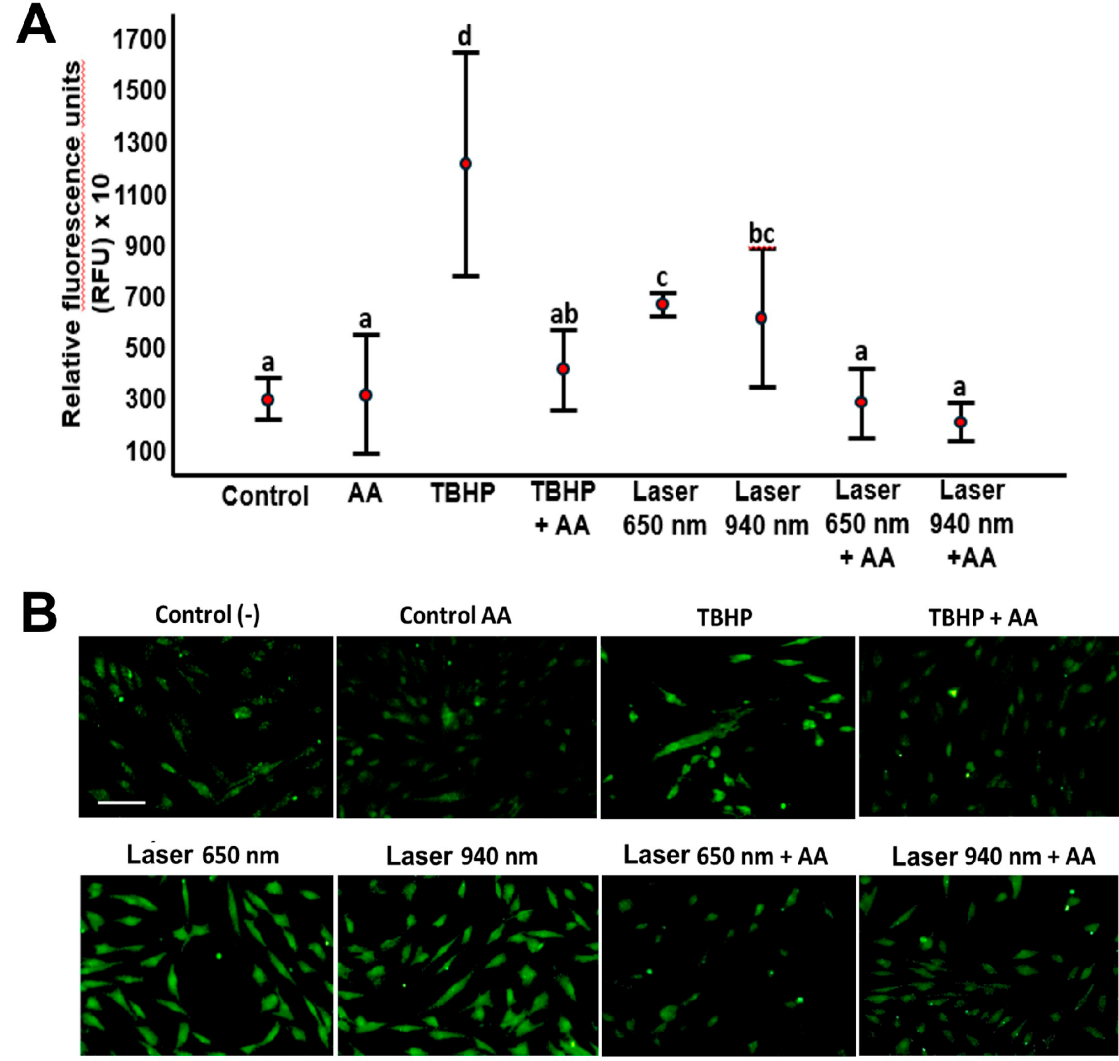
Effect of low-level laser therapy (LLLT) on reactive oxygen species (ROS) production in human gingival fibroblasts. ROS production was assessed using the dichlorofluorescein diacetate (DCF-DA) technique in cells irradiated with 650 nm and 940 nm lasers for 50 s. Each treatment was evaluated in relative fluorescence units (RFU) and quantified by spectrofluorometry. Panel (A) shows ROS levels in the positive control group (TBHP), negative control (ascorbic acid, AA), cells co-incubated with TBHP and AA, and cells irradiated with both laser wavelengths, with or without AA. Statistical differences among groups were determined using Tukey’s HSD test. Groups sharing the same lowercase letter are not significantly different, as follows: (**a**) Control, AA, TBHP + AA, Laser 650 nm + AA, and Laser 940 nm + AA; (**b**) TBHP + AA and Laser 940 nm; (**c**) Laser 650 nm and Laser 940 nm; and (**d**) TBHP, which differed significantly from all other groups. Different lowercase letters indicate statistically significant differences (*p* < 0.05). Panel (B) displays ROS detection by fluorescence microscopy (Axiovert 40 CFL, Carl Zeiss, USA) using a contrast setting of 5000, a range of 1.25, brightness of 14,215, and an exposure time of 90.2 ms. Scale bar: 100 μm

Increased reactive oxygen species (ROS) levels were observed in gingival fibroblasts exposed to both 650 nm and 940 nm lasers. Control cells without laser treatment exhibited low ROS levels. Exposure to TBHP at 1100X significantly increased ROS, which was reversed by 50 µg/mL ascorbic acid (AA). ROS production in fibroblasts after laser irradiation was significantly increased, regardless of the wavelength used (*P* < 0.05). The reduction of intracellular ROS by co-incubation with AA suggests that the antioxidant neutral*ise*s laser-induced ROS (Fig. 7A). Fluorescence microscopy confirmed increased ROS production in laser-exposed cells at both wavelengths, visual*ise*d as green fluorescence, compared to unexposed or AA-treated cells (Fig. 7B). These results indicate that increased intracellular ROS levels are a cellular response to LLLT.

**Fig. 8:**
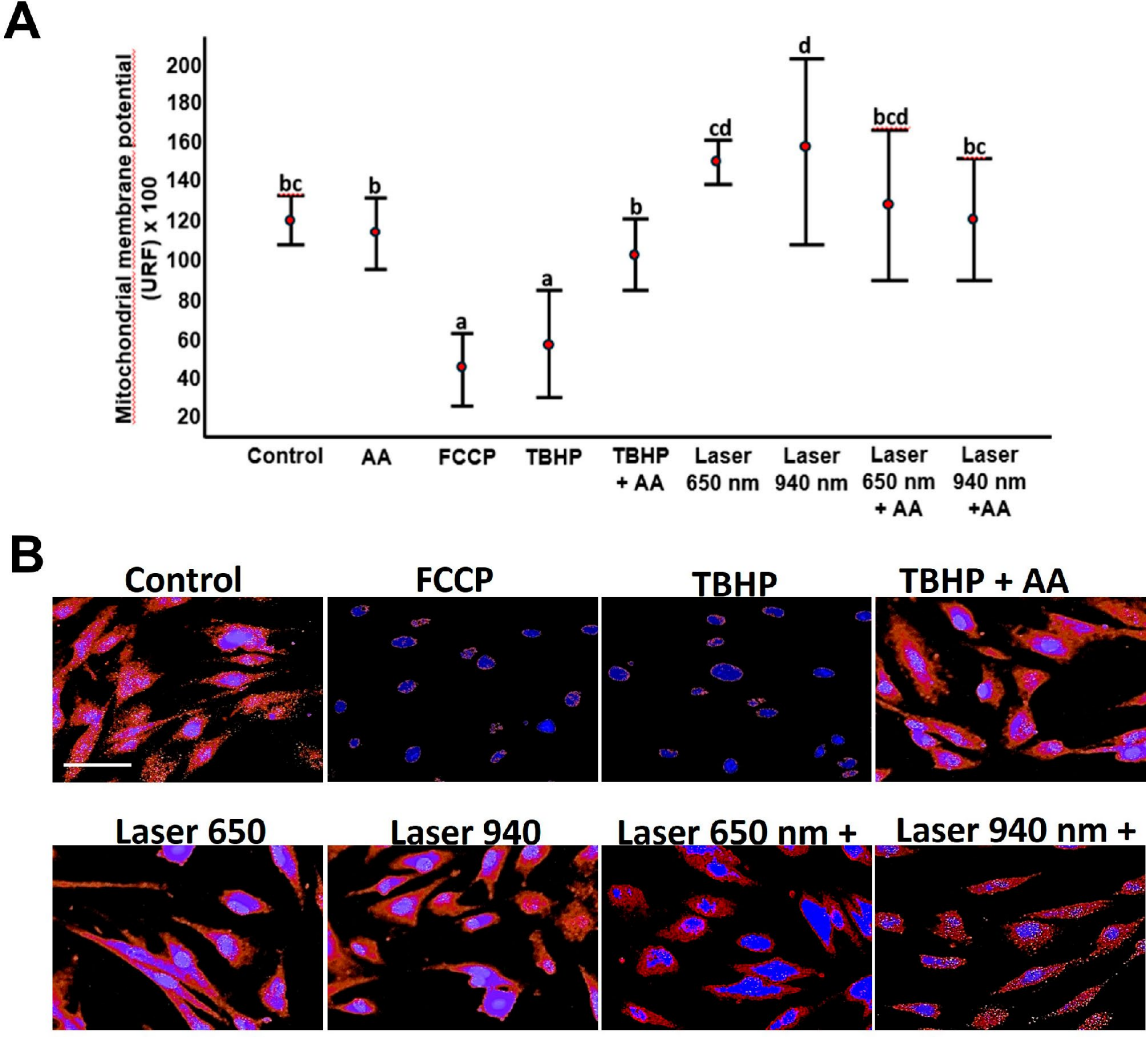
Mitochondrial membrane potential in laser-treated human gingival fibroblasts. Fluorescence intensity for each treatment was compared to the negative control (untreated cells), which was considered the baseline mitochondrial membrane potential (Δψm), and statistical differences between groups were analyzed using Tukey’s HSD test. The figure shows fluorescence intensity corresponding to the positive controls (FCCP and TBHP), as well as cells treated with each laser wavelength and co-incubated with 50 µg/mL of ascorbic acid (AA). Fluorescence was measured using a spectrofluorometer (Infinite M200, Tecan; Männedorf, Switzerland) at 549 nm excitation and 575 nm emission. Groups sharing the same lowercase letter are not significantly different, as follows: (**a**) FCCP and TBHP; (**b**) Control, AA, TBHP + AA, Laser 650 nm + AA, and Laser 940 nm + AA; (**c**) Control, Laser 650 nm, Laser 650 nm + AA, and Laser 940 nm + AA; and (**d**) Laser 650 nm, Laser 650 nm + AA, and Laser 940 nm. Different lowercase letters indicate statistically significant differences among groups (*p* < 0.05). FCCP and TBHP showed significant differences compared to all other experimental groups

Changes in mitochondrial membrane potential (Δψm) were assessed using the TMRM assay and spectrofluorometry. Cells exposed to AA showed no significant changes in Δψm compared to untreated controls. FCCP and TBHP at 1100X served as positive controls for uncoupling oxidative phosphorylation and inducing oxidative stress, respectively. Cells exposed to these positive controls showed a 63% (FCCP) and 52% (TBHP) decrease in Δψm due to impaired mitochondrial retention of TMRE staining. Evaluation of Δψm in TBHP- and AA-treated cells revealed a 37% restoration of potential, similar to untreated cells (Fig. 8). The data indicated that laser treatment induced a wavelength-independent increase in Δψm compared to the control group. Co-incubation with AA restored Δψm values across all exposure conditions.

## Discussion

LLLT has demonstrated photobiomodulatory effects on various cell types, inducing intracellular metabolic changes that activate physiological functions. Human gingival fibroblasts, the primary connective tissue cells, play a crucial role in extracellular matrix formation and gingival tissue homeostasis maintenance [12, 32].

This study evaluated the effects of PBM using LLLT on gingival fibroblast proliferation, viability, and ROS formation. We employed two wavelengths (650 nm and 940 nm) with a 50-second exposure time and 0.1 W output power. Post-irradiation effects on cell proliferation and number showed no significant differences between irradiated and non-irradiated cells during the first five days at either wavelength, using energy densities of 13.15 J/cm² (650 nm) and 6.4 J/cm² (940 nm). However, 10 days post-irradiation, both wavelengths induced a significant increase in cell proliferation, with no statistical differences between them. Cell viability remained consistent across all groups throughout the study. These findings suggest that LLLT induces a bio-stimulatory effect after 10 days of irradiation, regardless of the energy densities used.

Our results align with previous studies. Almeida-Lopes et al. [2] and Azevedo et al. [33] demonstrated that gingival fibroblasts cultured under stress conditions (5% FBS) or ideal conditions (10% FBS) exhibited similar or increased cell growth after PBM using wavelengths ranging from 660 nm to 786 nm with energy densities of approximately 2 J/cm², despite differences in power densities. Both studies concluded that stressed cells showed similar or higher cell growth after irradiation compared to cells cultured under ideal conditions.

Our cell proliferation findings support those of Pereira et al. [34], who observed a three- to six-fold increase in NIH-3T3 fibroblast numbers after irradiation with a 904 nm diode laser (energy densities of 3.0–5.0 J/cm²). Similarly, systematic reviews [35, 36] have reported increased gingival fibroblast proliferation following LLLT with wavelengths between 600 and 1000 nm and output powers ranging from 1 to 1000 mW. Conversely, Hakki and Bozkurt [37] noted no significant effects on fibroblast proliferation when using a 940 nm laser with high output powers, likely due to photothermal effects overriding PBM responses. This lack of effect could be attributed to the high output powers applied, which likely induced a predominantly photothermal effect rather than facilitating a PBM response.

Regarding cell viability, studies using wavelengths of 445 nm (energy densities between 4 and 6 J/cm²), 810–940 nm (100 mW, 0.5–2.5 J/cm²), and 810 nm (50 mW, 4 J/cm²) reported biostimulatory effects on fibroblast viability, proliferation, and migration [38–40]. However, for the 940 nm wavelength, and when combined with 810 nm, no significant differences in viability were observed, consistent with our findings. These variations may stem from differences in laser parameters, as insufficient energy below the physiological threshold may fail to elicit a response or even cause adverse effects [41–44].

The wavelength and exposure time are critical determinants of energy absorption in tissues, influencing the interaction between light and cellular structures. Variations in biostimulation effects observed across studies may be attributed to the use of different wavelengths, which can activate distinct chromophores, operate through varied mechanisms, and penetrate tissues to different depths [45, 46]. Exceeding the optimal laser dose may induce inhibitory effects, leading to cellular stress character*ise*d by elevated intracellular calcium levels, reduced cAMP and ATP production, decreased DNA synthesis, and increased expression of pro-apoptotic factors. Conversely, when the dose is within the optimal range, PBM occurs, resulting in enhanced cellular function and viability [47].

Morphological changes in fibroblasts were observed five days post-irradiation, with larger cytoplasmic extensions evident at 10 days, particularly under the 940 nm wavelength. Similar findings have been reported in previous studies, where gingival fibroblasts irradiated with diode lasers at wavelengths of 904 nm and 810 nm, with energy densities of 3 J/cm² and 15 J/cm², respectively, exhibited notable structural alterations. These changes, including modifications in mitochondria and endoplasmic reticulum, are thought to enhance collagen metabolism and contribute to the regenerative effects of PBM [7, 48, 49].

Treatment with secretome from gingival fibroblasts irradiated at wavelengths of 650 nm and 940 nm for 50 s demonstrated significant effects on cell migration and proliferation, beginning on day five post-irradiation and becoming more pronounced by day 10. A marked increase in cell proliferation was observed with secretomes irradiated at both wavelengths, with the most significant effect evident under 940 nm laser treatment. Fibroblasts are known to secrete essential extracellular matrix components, such as collagen types I and IV, fibronectin, laminin, proteoglycans, and hyaluronic acid, alongside lipids and extracellular vesicles [50]. These secretions contain biologically active paracrine factors critical for cellular communication and tissue regeneration [51]. These factors possess trophic, cytoprotective, and immunomodulatory properties, making them pivotal contributors to innovative regenerative strategies [14, 52].

Ahangar et al. (2020) [13] analysed the secretome of gingival fibroblasts, identifying various cytokines associated with inflammation, as well as growth factors like FGF-2 and VEGF, and accessory molecules. Their findings highlighted the ability of the secretome to promote wound healing by accelerating reepithelialisation, enhancing collagen production, and reducing inflammation in acute wound models in mice. Similarly, Bakopoulou et al. (2017) [14], isolated MSCs from gingival tissue and dental follicles, revealing that gingival-derived secretomes are enriched with genes linked to keratinisation, ectodermal development, and chemotaxis, which collectively support tissue repair and regeneration.

Despite these advancements, research on the secretome of cells treated with LLLT remains limited. Amaroli et al. (2022) [53] underscored the potential of PBM to enhance the secretome of MSCs, either by boosting the activity of young MSCs or rejuvenating older ones, ultimately improving their plasticity and regenerative capabilities. Investigating the composition and effects of the secretome from laser-irradiated gingival fibroblasts could provide critical insights into the mechanisms by which LLLT promotes cellular proliferation and migration, highlighting a promising area for further research.

Kocherova et al. (2021) [54], investigated the in vitro effects of LLLT at 635 nm and 808 nm on gingival fibroblasts (100 mW, 4 J/cm²) and analysed conditioned medium collected post-irradiation. Their findings revealed enhanced cell proliferation, modulation of oxidative stress, and reduced apoptosis, highlighting the potential of combining conditioned medium (secretome) with PBM to improve wound healing and support tissue engineering and regenerative medicine [12, 45]. Although our study used a single irradiation session, our results align with these findings, emphasizing the therapeutic potential of PBM.

The effects of LLLT on cell migration and wound closure were particularly significant at the 940 nm wavelength. This aligns with the findings of Hawkins and Abrahamse (2006) [55], who demonstrated laser-induced haptotaxis and chemotaxis accelerating wound healing in human skin fibroblast cultures using a 632 nm He-Ne laser with fluences ranging from 0.5 to 16 J/cm². Their study showed that fluences of 5 J/cm² notably stimulated proliferation, migration, and healing within a timeframe of 3 to 24 h. It is proposed that LLLT induces cell polar*isa*tion toward the wound site, thereby promoting directed cell movement and initiating the wound-healing process [55, 56].

Collagen production is essential for wound healing [56, 57] and is often used as a marker of fibroblast function. COL1A1 expression was markedly upregulated five days post-irradiation with 650 nm (2.7-fold increase) and 10 days post-irradiation with 940 nm. These findings suggest that near-infrared lasers may enhance collagen production and accelerate wound healing. Our results align with Frozanfar et al. (2013) [40], who observed increased COL1A1 expression in gingival fibroblasts three days after irradiation with an 810 nm laser at a fluence of 4 J/cm². Similarly, Yu et al. (2006) [58] demonstrated significant upregulation of collagen-related genes, including procollagen I α1, α2, and TGF, in gingival fibroblasts treated with a 585 nm laser at a fluence of 3 J/cm². However, contrasting results were reported by Pereira et al. (2002) [34] and Marques et al. (2004) [48], who found no effect on procollagen levels following irradiation with a 904 nm laser. These variations highlight the wavelength- and fluence-dependent effects of PBM on collagen synthesis, underscoring the importance of optimizing laser parameters for therapeutic applications.

Finally, ROS production increased with both wavelengths. ROS are generated following LLLT stimulation of specific chromophores, such as endogenous porphyrins or mitochondrial cytochromes. Upon light absorption, the redox activity of the respiratory chain accelerates, leading to ROS production [15]. Naderi et al. (2017) [46] reported that both 660 nm LLLT and LED light significantly increased ROS production in human skin fibroblasts (Hu02), activating the NF-κB pathway and influencing gene expression related to cell survival and proliferation. These findings suggest that PBM initiates biological processes by generating oxygen free radicals, with ROS levels serving as markers of energy uptake by intracellular receptors [15, 52, 56, 57].

At appropriate concentrations, ROS act as essential second messengers, regulating cellular processes such as proliferation, adhesion, migration, and survival [43]. Their effects vary by cell or tissue type, depending on enzymatic activity, signal transduction modulation, and DNA repair capacity [15]. However, excessive ROS levels can become cytotoxic, disrupting mitochondrial function and causing cellular damage [54, 59]. George et al. (2018) [45] found that an 825 nm laser was more effective in generating ROS than a 636 nm red laser. In contrast, our study did not observe significant differences in ROS production between the two wavelengths evaluated. Previous research has demonstrated that PBM with low-level lasers induces both ROS and antioxidant production, emphasizing the role of the cellular redox state in maintaining cellular activities [60–63].

## Conclusions

This study demonstrates that LLLT induces significant biological effects on human gingival fibroblasts, including enhanced cell proliferation, morphological changes, increased migration, and upregulated COL1A1 expression when treated with 650–940 nm wavelengths. These effects were associated with elevated ROS levels and mitochondrial activity, suggesting a positive impact on cellular metabolism and function. Furthermore, secretome derived from laser-irradiated cells stimulated proliferation and migration, highlighting the potential paracrine effects of LLLT.

The results indicate that the cellular response to LLLT depends on a precise combination of laser parameters, including wavelength, output power, energy density, irradiation time, and number of applications. Alterations in these parameters may influence the magnitude and nature of the observed effects. Notably, treatment with the 940 nm laser produced the most pronounced cellular changes compared to the 650 nm laser and non-irradiated controls, suggesting wavelength-specific advantages in therapeutic applications.

However, caution is advised when extrapolating these results to clinical settings. The in vitro nature of this study lacks the complexity of tissue interactions present in vivo, where multiple cell types and extracellular matrix components interact. Further research is necessary to validate these findings in more complex models and to optim*ise* LLLT parameters for clinical applications in tissue regeneration and wound healing.

In conclusion, while this study provides valuable insights into the effects of LLLT on gingival fibroblasts, additional investigations are required to fully elucidate the mechanisms underlying these effects and to determine their clinical relevance. Future studies should focus on translating these findings into practical therapeutic strategies for periodontal regeneration and wound healing.

## Data Availability

No datasets were generated or analysed during the current study.
